# Complete chloroplast genome of *Euphorbia micractina* Boiss (Euphorbiaceae: Euphorbia)

**DOI:** 10.1080/23802359.2022.2087557

**Published:** 2022-06-28

**Authors:** Dan Wang, Yu-Rou Dan, Ming Liu, Fu-Qiang Yin

**Affiliations:** a College of Biological and Food Engineering, Chongqing Three Gorges University, Chongqing, China; b The Chongqing Engineering Laboratory for Green Cultivation and Deep Processing of the Three Gorges Reservoir Area’s Medicinal Herbs, Chongqing, China

**Keywords:** Complete chloroplast genome, *Euphorbia micractina* Boiss, Euphorbiaceae, phylogenetic analysis

## Abstract

*Euphorbia micractina* Boiss is a plant with high medicinal value. Yet, its molecular biology is not fully understood. In this study, we sequenced the whole chloroplast genome (CP) sequence of *E. micractina* to study its phylogenetic relationship in Euphorbiaceae. The total length of the chloroplast genome of *E. micractina* is 162,056 bp, including a large single-copy (LSC) region of 89,936bp bp, a small single-copy (SSC) region of 18,376 bp, and a pair of identical inverted repeat regions (IRs) of 11,367 bp. The genome has 128 genes, including 84 protein-coding genes, 36 transfer RNA (tRNA) genes, and 8 ribosomal RNA (rRNA) genes. The overall GC content of the plastome is 35.7%. The phylogenetic analysis of *E. micractina* with 30 related species suggested a closest taxonomical relationship with *Euphorbia pekinensis* in the Euphorbiaceae family.

*Euphorbia micractina* Boiss is a plant that belongs to Euphorbiaceae and Euphorbia genus and is mainly distributed in Gansu, Qinghai, Sichuan, and Tibet, China. It was first described by Candolle in 1862 (Candolle 1862). The plant is commonly used to remove warts (Wu et al. 1991). So far, 16 terpenoids, 7 steroids, and 15 aromatic derivatives were isolated from the ethanol extract of *Euphorbia officinalis* (Tao et al. 2015, 2016). In 2012, halberylmethyl butane found in the genus *Euphorbia peplus* was approved by the American FDA to treat solar keratosis (Keating 2012). At present, the research on *Euphorbia officinalis* mainly focuses on its chemical components and pharmacological activities, while only a few studies have reported on its molecular biology. Therefore, in this study, we sequenced the whole chloroplast genome (cp) sequence of *Euphorbia micractina*, which may further promote the genetic research and resource utilization of this plant.

Fresh leaf materials of *E. micractina* were sampled from Xiaojin County, Aba Prefecture, Sichuan Province, located at 102°01′102.59″E, 30°35′31.43″N. The specimens were kept in the herbarium of the College of Biology and Food Engineering, Chongqing Three Gorges University (https://www.sanxiau.edu.cn/smkx/; contact person name: Nong ZHOU, Email: erhaizn@126.com) under the voucher number ZQ311416. Total genomic DNA was extracted from 100 mg of actively growing fresh leaves using a modified CTAB (hexadecyltrimethylammonium bromide) method, in which 4% CTAB was used instead of 2% CTAB, and adding approximately 1% polyvinyl polypyrrolidone (PVP) and 0.2% DL-dithiothreitol (DTT) (Yang et al. 2014) and sequenced with Illumina Hiseq 2500 (Novogene, Tianjing, China) platform. The cp genome was assembled using GetOrganelle (Jin et al. 2020) with *Euphorbia pekinensis* (NC_058897) as reference. Annotation was performed with the GeSeq (Tillich et al. 2017) and CpGAVAS2 (Shi et al. 2019). The complete chloroplast genome sequence of *E. micractina* was submitted to GenBank (accession number: OL622067).

The chloroplast genome of *E. micractina* has a typical quadripartite structure with a length of 162,056 bp, containing inverted repeats (IRs) of 11,367 bp separated by a large single-copy (LSC) region of 89,936 bp and a single small copy (SSC) region of 18,376 bp. The cpDNA contains 128 genes, comprising 84 protein-coding genes, 36 tRNA genes, and 8 rRNA genes. The overall GC content of the plastome is 35.7%.

To identify the phylogenetic position of *E. micractina,* phylogenetic analysis was performed based on complete cp genomes from 30 Euphorbiaceae species with *Daphniphyllum macropodum* and *Daphniphyllum oldhamii* as the outgroup species. The complete chloroplast genome sequences were aligned using MAFFT version 7 (Katoh and Standley 2013). Maximum likelihood (ML) analysis was performed with RAxML (Stamatakis 2014) based on the GTRGAMMA model using 1000 replicates of bootstrap analysis. The phylogenetic analysis showed a close relationship of *E. micractina* with *Euphorbia pekinensis* in the family of Euphorbiaceae (Figure 1). These newly characterized phylogenetic analysis fruits can be used to develop markers for further study on the phylogeny and evolution of the genus Euphorbia.

**Figure 1.**
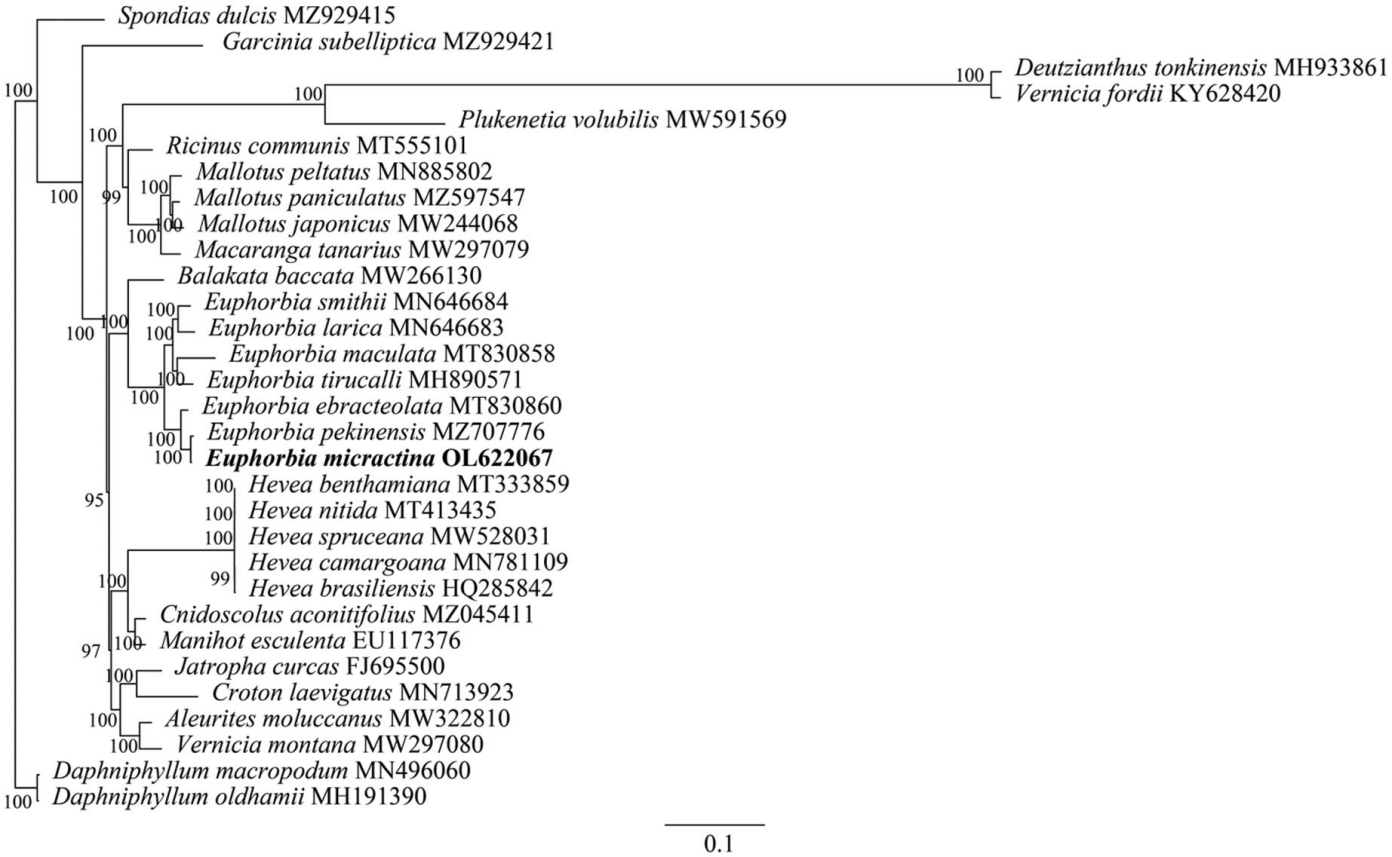
The construction of the phylogenetic tree performed using complete genomic sequences of 30 species and *E. micractina* based on the maximum likelihood method with a bootstrap value of 1000 replicates.

## Ethical approval

*Euphorbia micractina* Boiss is not a protected plant, and the current research will not cause any kind of damage to the population of *Euphorbia micractina* Boiss. Accordingly, no specific permissions are needed for this research.

## Author contributions

Dan Wang participated in the sample assembly and annotation work, write and revise the paper; Yu-rou Dan and Ming Liu were mainly responsible for the analysis and interpretation of data; Fu-qiang Yin was mainly responsible for the design of the experiment and approved the final version of the manuscript. All authors are accountable for all aspects of the work.

## Supplementary Material

Supplemental Material

## Data Availability

The data of this study are available in GenBank of NCBI at (https://www.ncbi.nlm.nih.gov) under accession no.OL622067. The associated BioProject, SRA, and BioSample numbers are PRJNA796732, SRR17604728, and SAMN24917951, respectively.
