# Supplementary material for: Complete chloroplast genome of Euphorbia micractina Boiss (Euphorbiaceae: Euphorbia)
Source: Mitochondrial DNA B Resour. 2022 Jun 28;7(6):1129–30. doi: 10.1080/23802359.2022.2087557 (PMC9246027; doi:10.1080/23802359.2022.2087557)
Supplement: Supplemental Material [file TMDN_A_2087557_SM1488.pdf]

MedSci

Editing By Professional Editors

CERTIFICATE OF ENGLISH EDITING

This document certifies that the manuscript entitled "Complete chloroplast genome of Euphorbia micractina Boiss (Euphorbiaceae: Euphorbia)" was proofread and edited for proper English language, grammar, punctuation, spelling, and overall style by one or more of the qualified scientific editors at MedSci, all of whom are native English speakers. Neither the research content nor the authors' intentions were altered in any way during the editing process.

Documents receiving this certification should be English-ready for publication; however, the author can accept or reject our suggestions and changes. To see the final MedSci edited version, please visit our verification page. If you have any questions or concerns about this document or certification, please contact us at editing@medsci.cn.

Corresponding Author:Fuqiang Yin

DATE:2022-02-21

SIGNATURE: MedSci

CODE:0221-FBD2-4F63-27D1-674B

This certificate may be verified at

https://editing.medscihealthcare.com/dist/medsci-order/#/verify
